# Pyoderma Gangrenosum: A Challenging Cutaneous Manifestation in Dubowitz Syndrome

**DOI:** 10.7759/cureus.43408

**Published:** 2023-08-13

**Authors:** Dewang B Ghode, Shoyeb Hirani, Sneha Kenjale, Arjun Heda, Sajid Hirani, Roshan Prasad, Mayur Wanjari

**Affiliations:** 1 General Surgery, Jawaharlal Nehru Medical College, Datta Meghe Institute of Higher Education and Research, Wardha, IND; 2 Medicine, Mahatma Gandhi Memorial (MGM) Medical College and Hospital, Aurangabad, IND; 3 Surgery, Jawaharlal Nehru Medical College, Datta Meghe Institute of Higher Education and Research, Wardha, IND; 4 Medicine, Jawaharlal Nehru Medical College, Datta Meghe Institute of Higher Education and Research, Wardha, IND; 5 Medicine and Surgery, Jawaharlal Nehru Medical College, Datta Meghe Institute of Higher Education and Research, Wardha, IND; 6 Research and Development, Jawaharlal Nehru Medical College, Datta Meghe Institute of Higher Education and Research, Wardha, IND

**Keywords:** underlying mechanisms, challenges, management, diagnosis, association, cutaneous manifestation, dubowitz syndrome, pyoderma gangrenosum

## Abstract

Pyoderma gangrenosum (PG) is a challenging cutaneous manifestation associated with Dubowitz syndrome, a rare genetic disorder characterized by multiple congenital anomalies, developmental delay, and distinctive facial features. This review article aims to provide a comprehensive overview of the association between Dubowitz syndrome and pyoderma gangrenosum, emphasizing the clinical presentation, challenges in diagnosis and management, and potential underlying mechanisms. A comprehensive literature search was conducted to gather relevant studies, and inclusion and exclusion criteria were applied to select appropriate articles. The association between Dubowitz syndrome and pyoderma gangrenosum has been documented in reported cases and studies. Clinical characteristics of Pyoderma gangrenosum in Dubowitz syndrome include painful necrotic ulcers with undermined borders. Diagnosing pyoderma gangrenosum in the context of Dubowitz syndrome can be challenging due to the overlapping clinical features and complexities associated with the syndrome. Managing pyoderma gangrenosum involves a multidisciplinary approach, with general principles of wound care, systemic therapy, and pain management. Specific considerations for treating pyoderma gangrenosum in Dubowitz syndrome include collaboration among specialists and careful monitoring. Future directions for management include further research to understand the underlying mechanisms and develop targeted therapies. Recognizing and addressing pyoderma gangrenosum in Dubowitz syndrome is crucial for optimal patient care. This review enhances awareness among healthcare professionals and provides insights for improving diagnosis, management, and treatment outcomes for individuals with this challenging combination of conditions.

## Introduction and background

Dubowitz syndrome is a rare genetic disorder characterized by multiple congenital anomalies, developmental delay, and a distinct facial appearance. It was first described by Victor Dubowitz in 1965 and has since been recognized as a complex condition with various clinical features. One of the challenging cutaneous manifestations associated with Dubowitz syndrome is pyoderma gangrenosum, an uncommon and often misdiagnosed skin disorder. This review article aims to provide an overview of Dubowitz syndrome, introduce pyoderma gangrenosum as a significant cutaneous manifestation of this syndrome, and discuss the purpose of this review [1-3].

Dubowitz syndrome primarily affects growth and development. It is characterized by a combination of features, including intrauterine growth retardation, short stature, microcephaly (abnormally small head circumference), distinctive facial features (such as a triangular face, wide-set eyes, and a small mouth), and intellectual disability. Other clinical findings may include skeletal abnormalities, immune system dysfunction, feeding difficulties, and increased infection susceptibility. The genetic basis of Dubowitz syndrome is not fully understood, although there is evidence of genetic and environmental factors contributing to its development [4].

Pyoderma gangrenosum is an uncommon and challenging skin disorder characterized by painful, necrotic ulcers with undermined borders. It is a neutrophilic dermatosis involving the abnormal accumulation and activation of neutrophils in the skin. The exact cause of Pyoderma gangrenosum is not well understood, but it is believed to be an autoimmune or autoinflammatory condition. Pyoderma gangrenosum can occur in isolation or be associated with various underlying systemic diseases, including inflammatory bowel disease, rheumatoid arthritis, and hematological malignancies [5].

This review article aims to highlight the association between Dubowitz syndrome and pyoderma gangrenosum, emphasizing the importance of recognizing and managing this cutaneous manifestation in patients with Dubowitz syndrome. Despite being a rare condition, Pyoderma gangrenosum can significantly impact the quality of life of affected individuals, and early recognition and appropriate management are crucial for optimal outcomes. By providing a comprehensive overview of Dubowitz syndrome, introducing Pyoderma gangrenosum as a challenging cutaneous manifestation, and discussing its diagnosis and management, this review aims to enhance awareness among healthcare professionals, facilitate early diagnosis, and improve the care provided to individuals with Dubowitz syndrome.

## Review

Methodology

A comprehensive literature search was conducted to gather relevant information for this review article. Multiple electronic databases, including PubMed, MEDLINE, and Google Scholar, were utilized with appropriate search terms related to Dubowitz syndrome and pyoderma gangrenosum. The search encompassed studies of all publication dates, including primary and review research articles. References from the identified articles were also reviewed for additional sources. The inclusion criteria for selecting studies included those examining the association between Dubowitz syndrome and pyoderma gangrenosum and providing clinical data on the condition's presentation, diagnosis, management, or outcomes in this context. Only studies published in peer-reviewed journals and available in English were considered. Exclusion criteria encompassed studies that did not specifically focus on pyoderma gangrenosum in Dubowitz syndrome, lacked relevance to the topic, were not in English, or were not published in peer-reviewed sources. The selection process involved screening titles and abstracts, followed by a full-text assessment of potentially relevant articles based on the inclusion and exclusion criteria. The final selection of studies formed the basis for the information presented in this review article. 

Dubowitz syndrome

Brief Description, Prevalence, and Demographics of Dubowitz Syndrome

Dubowitz syndrome is a rare genetic disorder characterized by a wide range of clinical features that affect multiple systems in the body. The exact prevalence of Dubowitz syndrome is unknown, but it is considered a rare disorder [2]. Dubowitz syndrome affects males and females equally, with no significant gender predilection. The prevalence of the syndrome is difficult to determine due to its rarity and the wide spectrum of clinical presentations. However, it has been estimated to occur in approximately 1 in 125,000 to 1 in 1,000,000 live births. Dubowitz syndrome has been reported in various ethnic groups and populations worldwide [3].

Key Clinical Features of Dubowitz Syndrome

Growth and developmental abnormalities are hallmarks of Dubowitz syndrome. Individuals with this condition typically display intrauterine growth retardation, which refers to impaired growth in the womb. Postnatally, affected individuals also experience growth deficiency, leading to short stature. Alongside these physical manifestations, developmental delay is commonly observed, and individuals may present with a spectrum of intellectual disabilities ranging from mild to severe [6]. These characteristics are well documented in the medical literature, so they serve as significant diagnostic criteria for Dubowitz syndrome.

Facial characteristics: The facial appearance of individuals with Dubowitz syndrome is often distinctive. It typically includes a triangular face shape with a high forehead and wide-set eyes. Other common facial features include a small mouth, a flat nasal bridge, and low-set ears. These facial characteristics contribute to the recognizable phenotype of Dubowitz syndrome [7].

Skeletal abnormalities: Skeletal manifestations are frequently seen in individuals with Dubowitz syndrome. Joint hypermobility, where the joints have an unusually large range of motion, is a common finding. Other skeletal abnormalities include clinodactyly, the presence of curved or bent fingers, and camptodactyly, a fixed flexion deformity of the fingers. Additionally, scoliosis, an abnormal spine curvature, may be present in some cases [8].

Immunological abnormalities: Individuals with Dubowitz syndrome often exhibit immune system dysfunction, which manifests as recurrent infections and increased susceptibility to respiratory tract infections. The immune system abnormalities contribute to the overall health challenges individuals with this syndrome face and require careful management [9].

Introduction to Cutaneous Manifestations in Dubowitz Syndrome

Cutaneous manifestations are common in individuals with Dubowitz syndrome and can vary widely in their nature and severity. These skin findings can include abnormalities such as dry skin, atopic dermatitis (eczema), increased susceptibility to infections, and delayed wound healing. In addition, rare cutaneous manifestations, such as Pyoderma gangrenosum, have been reported in association with Dubowitz syndrome. Pyoderma gangrenosum is a challenging and potentially devastating skin condition characterized by painful necrotic ulcers with undermined borders [10].

Understanding the cutaneous manifestations of Dubowitz syndrome, including Pyoderma gangrenosum, is crucial for the appropriate diagnosis, management, and comprehensive care of individuals with this complex genetic disorder. In the following sections, we will explore Pyoderma gangrenosum in more detail, including its epidemiology, pathogenesis, diagnosis, and treatment considerations within the context of Dubowitz syndrome [11].

Pyoderma Gangrenosum

Definition and Clinical Presentation of Pyoderma Gangrenosum

Pyoderma gangrenosum (PG) is a rare, non-infectious, neutrophilic dermatosis characterized by the development of painful, necrotic ulcers. The condition typically presents as single or multiple ulcers with undermined borders and a tendency to progress rapidly. The ulcers often have a purplish or violaceous appearance and can be deep and destructive, involving the dermis and sometimes even the underlying structures such as tendons or bones. PG lesions commonly occur on the lower extremities but can also affect other body areas, including the trunk, head, and neck [12].

Epidemiology and Prevalence of Pyoderma Gangrenosum

Pyoderma gangrenosum is a rare condition, and its exact prevalence is not well established. However, it is estimated to occur in approximately 3 to 10 cases per million people annually. PG can affect individuals of any age, but it most commonly presents in adults between 20 and 50. There is no known gender predilection for the condition [13].

Pathogenesis and Aetiology of Pyoderma Gangrenosum

The exact pathogenesis of Pyoderma gangrenosum is not fully understood. It is believed to be an immune-mediated disorder with underlying immune system dysregulation. The condition is characterized by the abnormal accumulation and activation of neutrophils, leading to tissue damage and the formation of ulcers. Various factors, including genetic predisposition, dysregulated inflammatory response, and environmental triggers, may contribute to the development of PG. It is often associated with other systemic diseases, including inflammatory bowel disease, rheumatoid arthritis, and hematological malignancies, suggesting a potential link between the pathogenesis of these conditions and PG [14].

Diagnosis of Pyoderma Gangrenosum

Diagnosing pyoderma gangrenosum can be challenging due to its variable presentation and similarity to other skin conditions. No specific diagnostic tests are available for PG, and the diagnosis is primarily based on clinical features and the exclusion of other potential causes of similar ulcerative skin lesions. The diagnosis is often made through clinical evaluation, medical history assessment, physical examination, and the exclusion of infectious, vasculitic, and malignant etiologies. An ulcer biopsy is usually performed to rule out other causes and assess for characteristic histopathological features, including neutrophilic infiltrates [15].

Differential Diagnosis of Pyoderma Gangrenosum in Dubowitz Syndrome

In individuals with Dubowitz syndrome, the presence of pyoderma gangrenosum adds a layer of complexity to the diagnosis. Differential diagnosis of PG in the context of Dubowitz syndrome should consider other cutaneous manifestations commonly seen in Dubowitz syndrome, such as atopic dermatitis, delayed wound healing, and increased susceptibility to infections. It is essential to consider other potential causes of ulcers, including infectious etiologies, vasculitic conditions, and malignancies. Close collaboration between dermatologists, geneticists, and other specialists is crucial to reaching an accurate diagnosis and providing appropriate management for patients with Dubowitz syndrome and suspected or confirmed pyoderma gangrenosum [11].

Association between Dubowitz syndrome and pyoderma gangrenosum

Overview of Reported Cases and Studies

The association between Dubowitz syndrome and pyoderma gangrenosum has been documented in several reported cases and studies. Although rare, the occurrence of pyoderma gangrenosum in individuals with Dubowitz syndrome highlights an important cutaneous manifestation that can significantly impact patient management and quality of life. Published case reports, and retrospective studies have contributed to our understanding of this association, shedding light on the clinical characteristics, challenges in diagnosis, and potential underlying mechanisms [16].

Clinical Characteristics of Pyoderma Gangrenosum in Dubowitz Syndrome

Individuals with Dubowitz syndrome may present with unique clinical characteristics. The onset of pyoderma gangrenosum in Dubowitz syndrome can vary, ranging from infancy to adulthood. The ulcers may develop spontaneously or after minor trauma. They tend to be deep and painful and exhibit a progressive course. Lesions often have undermined borders and may show a predilection for certain anatomical sites, such as the lower extremities. However, the clinical presentation can be variable, and the association between Dubowitz syndrome and pyoderma gangrenosum needs further exploration to establish a more comprehensive understanding of its specific clinical features (Table 1) [11].

**Table 1:** Clinical characteristics of pyoderma gangrenosum in Dubowitz syndrome The author has created the table.

Clinical Characteristics	Description
Skin Ulcers	Painful, necrotic, and rapidly progressing skin ulcers with irregular borders and often undermined edges. Ulcers may be deep and can affect any part of the body.
Distribution	Lesions may appear in various areas, including the extremities, trunk, face, and perineal region. The distribution may be asymmetric and involve multiple sites simultaneously.
Extracutaneous Involvement	Pyoderma gangrenosum may be associated with extracutaneous manifestations, including arthritis, inflammatory bowel disease (IBD), and ocular inflammation.
Pathergy Phenomenon	Skin lesions may develop or worsen at the site of trauma or injury, indicating an exaggerated and abnormal response to minor trauma. This feature helps distinguish pyoderma gangrenosum from other ulcerative conditions.
Recurrent Course	Recurrent flares and remissions often characterize the disease course. Sudden exacerbations may follow periods of quiescence without apparent triggers.
Pain and Discomfort	Patients typically experience significant pain and discomfort due to the nature of the skin ulcers and their association with underlying systemic conditions.
Chronicity	Pyoderma gangrenosum in Dubowitz syndrome can be a chronic and persisting condition requiring long-term management and care.
Association with Dubowitz Syndrome	The co-occurrence of pyoderma gangrenosum with Dubowitz syndrome suggests potential underlying genetic and immune system links between the two conditions.
Diagnosis Challenges	Diagnosing pyoderma gangrenosum in the context of Dubowitz syndrome may be challenging due to overlapping clinical features with other conditions and the complexity of managing multiple comorbidities.
Response to Treatment	The response to conventional therapies such as corticosteroids and immunosuppressants may vary. Some patients may require specialized and individualized treatment approaches.

Challenges in Diagnosing Pyoderma Gangrenosum in Dubowitz Syndrome

Diagnosing pyoderma gangrenosum in individuals with Dubowitz syndrome can pose unique challenges. Multiple congenital anomalies and clinical complexities associated with Dubowitz syndrome may obscure the recognition of pyoderma gangrenosum as a distinct cutaneous manifestation. The differential diagnosis of ulcers in individuals with Dubowitz syndrome should consider various factors, including other cutaneous manifestations of the syndrome, delayed wound healing, and increased susceptibility to infections. The lack of specific diagnostic tests for pyoderma gangrenosum further complicates the diagnosis, necessitating a thorough evaluation and collaboration between dermatologists and other specialists to ensure accurate identification and appropriate management [5].

Potential Underlying Mechanisms for the Association

The exact underlying mechanisms linking Dubowitz syndrome and pyoderma gangrenosum remain unclear. However, several hypotheses have been proposed. It is speculated that the genetic and immune dysregulation observed in Dubowitz syndrome may contribute to the development of pyoderma gangrenosum. The abnormal immune response and neutrophil dysfunction seen in pyoderma gangrenosum could be influenced by genetic factors associated with Dubowitz syndrome. Additionally, systemic inflammation, such as in inflammatory bowel disease or other comorbidities commonly associated with pyoderma gangrenosum, may also play a role. Further research is needed to unravel the precise pathophysiological mechanisms underlying the association between Dubowitz syndrome and pyoderma gangrenosum [17].

Continued investigation into the clinical and molecular aspects of this association will aid in deepening our understanding of both conditions and pave the way for improved diagnostic approaches, management strategies, and potential targeted therapies. In the subsequent sections, we will explore the diagnosis, management, and treatment options for pyoderma gangrenosum in the context of Dubowitz syndrome, incorporating relevant case studies and examples [18].

Management and treatment

General Principles of Managing Pyoderma Gangrenosum

Wound care: Proper wound care prevents infection and facilitates healing. This involves regular cleansing of the ulcer to remove debris and bacteria, debridement of necrotic tissue, and applying appropriate dressings to promote a moist wound environment [19].

Systemic therapy: Systemic medications are often necessary to control the underlying inflammatory process in pyoderma gangrenosum. Corticosteroids are commonly used as a first-line treatment to suppress inflammation. In cases where corticosteroids are not effective or are not well tolerated, other immunosuppressive agents such as azathioprine or mycophenolate mofetil may be prescribed. Biologic agents, such as anti-TNF-alpha agents like infliximab or adalimumab, have also shown efficacy in severe or refractory cases [20].

Topical therapy: Topical treatments can be employed for localized lesions or to aid in healing smaller ulcers. Corticosteroid creams or ointments may be applied directly to the affected area to reduce inflammation and promote healing. Other topical agents may be utilized, such as calcineurin inhibitors (e.g., tacrolimus) or wound healing-promoting dressings [21].

Pain management: Pyoderma gangrenosum ulcers can be extremely painful, and effective pain control is essential to improving the patient's quality of life. Analgesics, such as nonsteroidal anti-inflammatory drugs (NSAIDs) or opioids, may be prescribed to manage pain. Local anesthetic creams or patches can also relieve localized pain [22].

Specific Considerations for Treating Pyoderma Gangrenosum in Dubowitz Syndrome

Collaboration with specialists: Given the multi-system involvement in Dubowitz syndrome, close collaboration among specialists is essential. This may include dermatologists, geneticists, immunologists, and other relevant healthcare professionals. By working together, these specialists can provide comprehensive and coordinated care, considering the diverse aspects of the syndrome and its associated manifestations [23].

Tailored treatment approach: The treatment approach for Pyoderma gangrenosum in individuals with Dubowitz syndrome should be individualized. It should consider the patient's overall health, specific clinical characteristics, and potential comorbidities associated with Dubowitz syndrome and pyoderma gangrenosum. Each patient's unique circumstances and needs should guide the selection of treatment modalities [24].

Careful monitoring: Individuals with Dubowitz syndrome may experience delayed wound healing and increased infection susceptibility. Therefore, careful and regular monitoring of the ulcer and the patient's overall clinical status is crucial. This involves close observation of the ulcer's progress, assessing for any signs of complications or deterioration, and ensuring timely intervention when necessary. Prompt recognition and management of any issues can help prevent further complications and promote optimal healing [25].

Case Studies or Examples of Treatment Approaches

Case studies describing successful management strategies: These case studies focus on specific patients with both Dubowitz syndrome and pyoderma gangrenosum. They highlight successful treatment approaches that have led to controlling pyoderma gangrenosum ulcers. Examples may include systemic immunosuppressive agents such as corticosteroids, azathioprine, or mycophenolate mofetil. Additionally, case studies may explore biological therapies, such as anti-TNF-alpha agents, which have shown promise in managing pyoderma gangrenosum [11,26].

Illustrations of wound care techniques: These examples demonstrate the selection and application of appropriate dressings and wound care techniques for individuals with pyoderma gangrenosum in the context of Dubowitz syndrome. They may outline the use of advanced dressings, such as hydrocolloids or foam dressings, to promote wound healing and provide a moist wound environment. Additionally, illustrations may highlight adjunctive therapies, such as negative pressure wound therapy (NPWT), which can aid in wound closure and tissue regeneration [27].

Reports on multidisciplinary approaches emphasize the importance of a collaborative approach involving various specialists. Dermatologists, geneticists, immunologists, and other experts work together to develop tailored treatment plans and long-term management strategies for individuals with Dubowitz syndrome and pyoderma gangrenosum. These reports provide insights into the comprehensive care provided to address the complex needs of patients, considering the underlying genetic factors, immune dysregulation, and potential comorbidities [28].

Potential Future Directions for Management

Elucidating the underlying pathophysiology: Future research should focus on conducting comprehensive studies to unravel the precise pathophysiological mechanisms linking pyoderma gangrenosum and Dubowitz syndrome. Investigating the genetic factors and immune dysregulation specific to Dubowitz syndrome that contribute to the development of pyoderma gangrenosum will be crucial in identifying potential therapeutic targets.

Identification of biomarkers: There is a need to identify reliable and specific biomarkers associated with the onset and progression of pyoderma gangrenosum in individuals with Dubowitz syndrome. Biomarkers could aid in early diagnosis, prognosis, and treatment monitoring, ultimately improving patient outcomes.

Targeted therapies: Developing targeted therapeutic agents tailored to the unique needs and characteristics of individuals with both Dubowitz syndrome and pyoderma gangrenosum holds promise for more effective management. Investigating immune-modulating therapies or molecular-based interventions specific to this patient population should be explored.

Role of immune modulation: Further investigation into the role of immune dysregulation in developing and exacerbating pyoderma gangrenosum in Dubowitz syndrome patients may uncover potential interventions to control the inflammatory response and mitigate skin ulceration.

Clinical trials: Conducting well-designed clinical trials focusing on patients with both Dubowitz syndrome and pyoderma gangrenosum is essential to evaluating the safety and efficacy of new interventions. Randomized controlled trials comparing different treatment modalities will provide valuable evidence to guide clinical decision-making.

Long-term outcomes and quality of life: Under various management strategies, research should assess the long-term outcomes and quality of life of individuals with Dubowitz syndrome and pyoderma gangrenosum. Understanding the impact of treatment on patients' physical, psychological, and social well-being will contribute to more patient-centered care.

Multidisciplinary collaboration: Facilitating collaboration among healthcare professionals, including dermatologists, geneticists, immunologists, and other specialists, will foster a holistic approach to managing this complex condition. Multidisciplinary teams can exchange knowledge, share best practices, and develop comprehensive treatment plans.

Patient registries: Establishing patient registries to collect and analyze data on individuals with Dubowitz syndrome and pyoderma gangrenosum can provide valuable insights into disease prevalence, progression, and treatment responses. These databases can serve as a foundation for future research and evidence-based guidelines.

## Conclusions

In conclusion, pyoderma gangrenosum (PG) represents a significant challenge in the context of Dubowitz syndrome, a rare genetic disorder characterized by multiple congenital anomalies, developmental delay, and distinctive facial features. This review has highlighted the critical importance of recognizing and managing PG in individuals with Dubowitz syndrome, considering its potential impact on the quality of life of affected individuals. The characteristic features of PG in this syndrome, such as the development of painful, necrotic ulcers with undermined borders, further complicate the diagnosis process. Given the overlapping clinical features and complexities associated with Dubowitz syndrome, a multidisciplinary approach involving dermatologists, geneticists, and other specialists is crucial to achieving an accurate diagnosis and implementing tailored treatment strategies. The management of PG in the context of Dubowitz syndrome should build upon the general principles of PG management, encompassing wound care, systemic therapy, and pain management. However, it is imperative to adapt these approaches to suit the unique challenges posed by this genetic disorder. Collaborating with specialists and maintaining careful monitoring of ulcers are vital components of the treatment plan. Looking ahead, further research is warranted to gain a deeper understanding of the underlying mechanisms connecting Dubowitz syndrome and PG. Such research efforts can pave the way for the development of targeted therapies that would improve the prognosis and overall care for individuals grappling with this intricate combination of conditions. Ultimately, recognition and timely intervention for PG in Dubowitz syndrome are of utmost importance to enhance patient outcomes and improve the overall quality of life for those affected by this complex medical scenario. By fostering collaboration among healthcare professionals and advancing research efforts, we can strive to make substantial progress in managing and ultimately preventing the adverse effects of PG in Dubowitz syndrome.
